# Effect of Chinese Medicine Xinmaitong on Blood Pressure in Spontaneously Hypertensive Rats

**DOI:** 10.1155/2020/7869403

**Published:** 2020-12-18

**Authors:** Bin Zhang, Dong Li, Gexiu Liu, Wenfeng Tan, Jun Guo, Gaoxing Zhang

**Affiliations:** ^1^Department of Cardiovascular Disease, Jiangmen Central Hospital, Affiliated Jiangmen Hospital of Sun Yat-sen University, Jiangmen 529030, China; ^2^Department of Cardiology, First Affiliated Hospital of Jinan University, Guangzhou 510630, China; ^3^Department of Intensive Care Unit, Jiangmen Central Hospital, Affiliated Jiangmen Hospital of Sun Yat-sen University, Jiangmen 529030, China; ^4^Clinical Experimental Center, Jiangmen Central Hospital, Affiliated Jiangmen Hospital of Sun Yat-sen University, Jiangmen 529030, China; ^5^Institute of Hematology, Medical College, Jinan University, Guangzhou 510632, China

## Abstract

**Objective:**

To investigate the effect of traditional Chinese antihypertensive compound Xinmaitong on blood pressure and vasoactive factors of vasoconstrictor endothelin-1 (ET-1) and vasodilator calcitonin gene related peptide (CGRP) in spontaneously hypertensive rats (SHRs) with early stage hypertension.

**Methods:**

Twenty male SHRs were randomly divided into two groups: 10 for hypertensive control group and 10 for hypertensive treatment group. In addition, 10 Wistar rats were used as the normal control group without any intervention. SHRs of hypertensive treatment group were orally treated with Xinmaitong, while the hypertensive control group was treated with the normal saline (NS) for a total of eight weeks. The blood pressure in SHRs was examined before and after the end of the eight-week study. After treatment, the rats were killed and the blood samples were collected to measure plasma levels of ET-1 and CGRP by ELISA method, respectively. Meanwhile, the aorta rings were isolated for measuring the mRNA expression of ET-1 and CGRP by PCR. Moreover, the protein levels of ET-1 and CGRP were studied by immunohistochemical.

**Results:**

Daily oral administration of Xinmaitong resulted in significant fall in the SHRs' blood pressure, including systolic and diastolic blood pressures (SBP and DBP), mean blood pressure (MBP), and pulse pressure (PP). The plasma ET-1 levels were reduced and CGRP increased. In parallel, the mRNA and protein expression of ET-1 were decreased, whereas the mRNA and protein expression of CGRP were enhanced in SHRs treated with Xinmaitong.

**Conclusion:**

The present study demonstrated for the first time that Xinmaitong leads to the fall in blood pressure of SHRs and that this antihypertensive effect is, at least in part, due to improvement of arterial tone.

## 1. Introduction

Hypertension continues to be a classic worldwide problem and a major global health burden. Hypertension (HTN) or prehypertension (PreHTN) alone combined with other metabolic diseases such as obesity and diabetes is one of the major risk factors for the pathogenesis of atherosclerotic cardiovascular disease (ASCVD) [1, 2]. PreHTN, the intermediate stage between HTN and normal blood pressure, is associated with subclinical atherosclerosis and target-organ damage [3]. PreHTN and HTN pose significant clinical and public health challenges for both economically developing and developed nations. Reduced vasodilator [4] as well as increased vasoconstrictor [5] is the hall marker of hypertensive vascular injury. Therefore, effective blood pressure-lowering intervention together with the balance of vasoactive materials towards enhanced production of vasodilator has significant clinical implication in order to prevent and treat ASCVD.

Xinmaitong is a traditional Chinese medicine compound preparation consisting of *Angelica sinensis*, *Salvia miltiorrhiza*, Uncaria Chinensis, *Panax notoginseng*, cassia seed, *Pueraria lobata*, *Sophora pubescens*, Mao Holly, *Prunella vulgaris*, and *Achyranthes bidentata*, which were usually used for antihypertension and has been also used to treat hypertension and ASCVD patients [6–13]. Clinical applications and experimental studies have shown that it has a significant effect on myocardial ischemia injury; in addition, clinical studies have also confirmed that Xinmaitong can improve the elastic index of large and small arteries and reduce the hypersensitivity CRP in patients with coronary heart disease. Another study shown that candesartan combined with Xinmaitong has a higher control rate in patients with simple diastolic hypertension. This suggests that Xinmaitong is an effective drug that can effectively protect the function of vascular endothelial cells and may play an antihypertensive effect [6, 14–18].

Calcitonin gene related peptide (CGRP) is one of the strongest vasodilators ever known, with the effects of lowering blood pressure, lowering peripheral resistance, diastolic renal arteries, and significantly increasing renal blood flow. CGRP also has a strong diastolic effect on the coronary arteries, and it is also effective on the coronary arteries of atherosclerosis. This diastolic effect does not depend on the presence of vascular endothelium, and it is not affected by serotonin receptor blockers. This indicates that CGRP binds to a specific CGRP receptor [19–22]. However, no study was performed to investigate the effects of Xinmaitong on blood pressure and vasoactive materials in spontaneous hypertensive rats (SHRs). Therefore, the present study was designed to observe the impact of Xinmaitong on blood pressure and vasoactive factors of vasoconstrictor endothelin-1 (ET-1) and vasodilator calcitonin gene related peptide (CGRP) in spontaneously hypertensive rats (SHRs).

## 2. Materials and Methods

Twenty male SHRs and 10 Wistar rats, aged four weeks and weighed 140–150 g, were purchased (Vital River Laboratories, Charles River Company, Beijing, China). All rats were housed in controlled temperature (23 to 25°C) and lighting (8:00 AM to 8:00 PM light; 8:00 PM to 8:00 AM dark) and had free access to standard food and drinking water. All animal experiments were approved by the Administrative Committee of Experimental Animal Care and Use of our hospital and conformed to the National Institute of Health guidelines on the ethical use of animals.

### 2.1. Experimental Protocol

Chinese herbal compound Xinmaitong was provided by Guizhou Yibai Pharmaceutical Co., Ltd. SHRs were bred in the Experimental Animal Center, Medical School of Sun-Yat Sen University, Guangzhou, China. After they were bred for seven days of adaptation, 20 SHRs were randomly divided into 2 groups: 10 for SHR control (SHR-C) group and 10 for SHR Xinmaitong (SHR-X) group. The 10 Wistar-Kyoto (WKY) rats were used as the normal control group. The WKY was fed without any intervention, The SHR-X were administered by gavage with 10 ml/kg body weight of 4.536% Xinmaitong suspension. The dosage of the drug was converted with the amount of the clinical routine drug (Xinmaitong 72 mg/kg) with reference to the conversion factor 6.3 between rats and humans. Furthermore, in consideration of rat generally administration volume is 1 ml/100 g, we choose dose of Xinmaitong at 10 ml/kg of body weight of 4.536% Xinmaitong suspension. In order to reduce the variability of the difference between the two groups, the SHR-C group was administered 10 ml/kg of body weight of 0.9% NS at the same time. The intervention time lasted eight weeks.

After eight weeks of intervention, all of the rats were killed and blood samples were harvested from the rats to test the plasma levels of ET-1 and CGRP. The aortas of the rats were isolated for the PCR and immunohistochemistry.

### 2.2. Biochemical Measurement

To observe the safety and side effects of Xinmaitong therapy, after eight weeks of intervention, all of the rats were killed and blood samples were harvested from the rats to measure hepatic and renal functions of the rats such as AST/GOT, ALT/GPT, TP, TBA, UREA, BUN, and CREA. The kits for these parameters were provided by Nanjing Jiancheng Bioengineering Institute, China.

### 2.3. Blood Pressure Measurement

To demonstrate the beneficial effect of Xinmaitong treatment on arterial blood pressure, we used a special sphygmomanometer called BP98A intelligent noninvasive blood pressure monitor to measure the rat's tail artery blood pressure (TABP) according to the machine manual. The first step is to open the device and software. The second step is to fix the rat so that it cannot move and fix the tail artery detector to the rat's tail. The third step is to keep the rat in a calm state and judge it to be in a stable state according to the software waveform. If the waveform is unstable, the measurement is delayed until the waveform is stable. The fourth step is to start the measurement, repeat the measurement three times, and take the average value. In order to ensure the accuracy of the measurement and to handle stress of animals, the measurement is guaranteed to be performed by the same operator in the same time period and environment. In addition, all rats had a two to three days' adaption measurement test before each measurement was performed. Furthermore, we take the performance three times only when the software shows the blood flow of the rat is stable; if the blood flow is unstable, the stabilization time can be appropriately extended and measured after the blood flow is stable.

Before and at the termination of eight-week treatment, all rat's TABP were measured by the same researcher. The systolic blood pressure (SBP), diastolic blood pressure (DBP), mean blood pressure (MBP), and pulse pressure (PP) were recorded.

### 2.4. ET-1 and CGRP Measurement

To evaluate the impact of Xinmaitong on vascular function, the plasma levels of vasoconstrictor ET-1 and vasodilator CGRP were examined. The ELISA kits were provided by Uscn Life Science Inc. Wuhan and we did the test according to the manufacturer's instructions.

### 2.5. PCR of ET-1 and CGRP

The thoracic aortas of the rats were isolated, and the isolated blood vessel was cut into three pieces of 3–4 mm wide vascular rings. Determination of thoracic aorta ET-1and CGRP mRNA were detected by PCR while the protein expression of them were examined by immunohistochemistry. A semiquantitative determination was carried out with a gelatin image analyzer, and the relative density grey value of ET-1and CGRP was used to stand for the relative expression quantity of ET-1, CGRP mRNA, and protein.

### 2.6. Immunohistochemistry of ET-1 and CGRP

Tissue sections were prepared of the thoracic aortas after fixation in 4% paraformaldehyde, dehydration, and embedding in paraffin. The expression of ET-1 and CGRP in the aorta tissue was examined using the SP immunohistochemistry kit according to the manufacturer's instructions. Densitometric analysis of immunocytochemical staining of ET-1and CGRP was carried out, and ET-1and CGRP staining intensity was expressed in optical density (OD) units.

### 2.7. Statistical Analysis

All the data were expressed as mean ± standard deviation and analyzed with the Statistical Package for the Social Sciences version 12.0 (SPSS 12.0). Comparisons between groups or between pre- and posttreatment were performed by *t* -test. The persons who analyzed the data were blinded to treatment-group assignment. For the graph made, Graphpad Prism 5 software was used. Throughout this study, a *P*-value less than 0.05 was considered statistically different.

## 3. Results

### 3.1. Safety Evaluation

The body weight and food intake of three groups were recorded before and after intervention. After eight weeks of treatment, the hearts of the rats were harvested. Weight of rats were measured and compared. For further safety and side effects evaluation, AST/GOT, ALT/GPT, TP, TBA, UREA, BUN, CREA, and *γ*-GT/*γ*-GTT were tested as the indicators for hepatic and renal function. Results show there were no significant differences in all the previously mentioned parameters in rats between the two groups (*P* > 0.05, Table 1).

### 3.2. Effects on SBP, DBP, MABP, and PP

**Figure 1:**
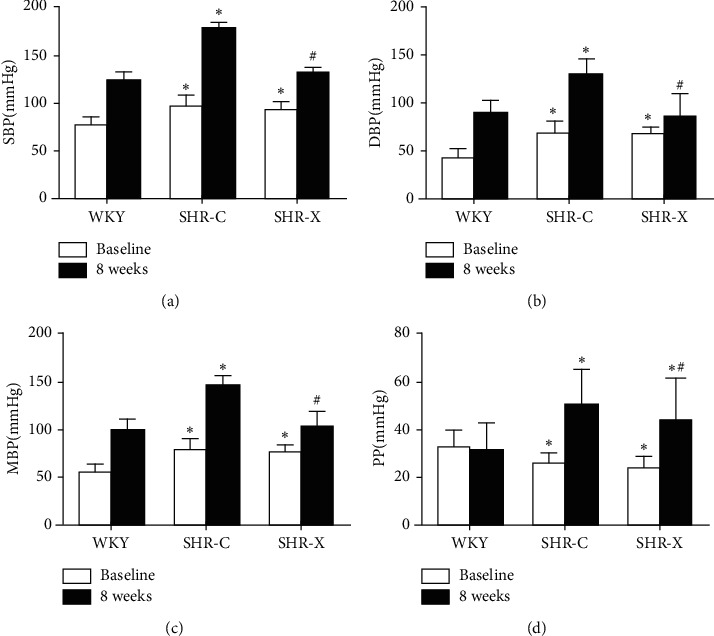
The effect of Xinmaitong on the blood pressure. Values are means ± SD. WKY: Wistar-Kyoto rats; SHR: spontaneously hypertensive rats; SHR-C: SHR control; SHR-X: SHR Xinmaitong treatment. ^*∗*^*P* < 0.05 versus WKY. ^#^*P* < 0.05 versus SHR-C.

SBP, DBP, MABP, and PP had no significant differences before treatment between SHR-C and SHR-X groups (*P* > 0.05) while they showed statistically significant differences after treatment (SBP lowering 46 mmHg, DBP lowering 41 mmHg, MABP lowering 42 mmHg, PP lowering 6 mmHg, *P* < 0.05, Figure 1). This indicates that Xinmaitong had an antihypertensive effect, including SBP, DBP, MABP, and PP.

### 3.3. Impact on Vasoconstrictor ET-1 and Vasodilator CGRP

**Figure 2:**
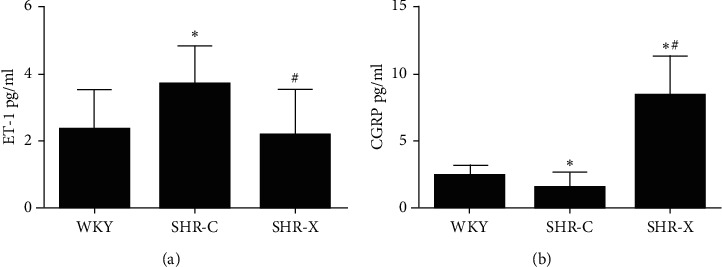
The effect of Xinmaitong on the blood vasoactive materials ET-1 and CGRP. Values are means ± SD. WKY: Wistar-Kyoto rats; SHR: spontaneously hypertensive rats; SHR-C: SHR control; SHR-X: SHR Xinmaitong treatment; ET-1: endothelin-1; CGRP: calcitonin gene-related peptide. ^*∗*^*P* < 0.05 versus WKY. ^#^*P* < 0.05 versus SHR-C.

After eight weeks of treatment, the content of vasoconstrictor endothelin-1 (ET-1) in the SHR-C group was higher than that of WKY and SHR-X groups, while the content of vasodilator calcitonin gene related peptide (CGRP) in SHR-C group was lower than that of WKY and SHR-X groups, showing a statistically significant difference (*P* < 0.05, Figure 2).

### 3.4. Comparison of the mRNA and Protein Expression Levels of ET-1 and CGRP

**Figure 3:**
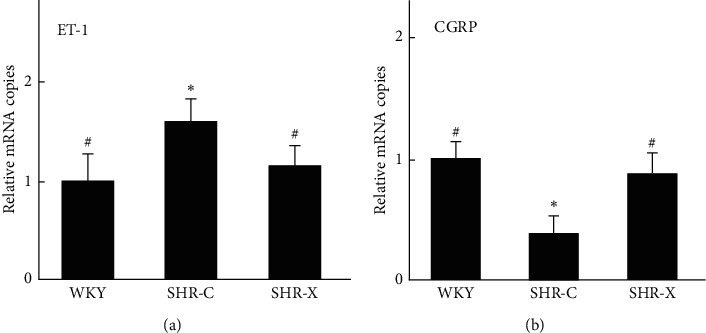
The level of ET-1 and CGRP mRNA. Values are means ± SD. WKY: Wistar-Kyoto rats; SHR: spontaneously hypertensive rats; SHR-C: SHR control; SHR-X: SHR Xinmaitong treatment; ET-1: endothelin-1; CGRP: calcitonin gene-related peptide. ^*∗*^*P* < 0.05 versus WKY. ^#^*P* < 0.05 versus SHR-C.

**Figure 4:**
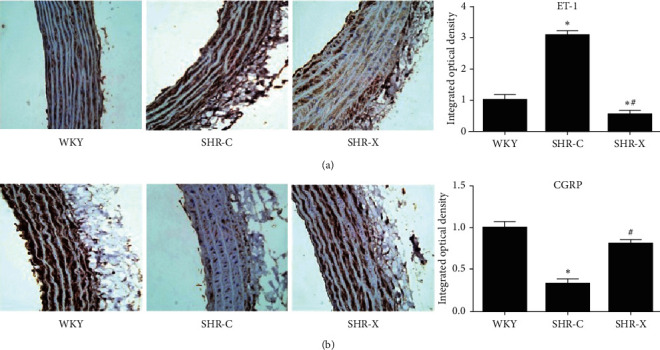
Immunohistochemistry of ET-1 and CGRP. Values are means ± SD. WKY: Wistar-Kyoto rats; SHR: spontaneously hypertensive rats; SHR-C: SHR control; SHR-X: SHR Xinmaitong treatment; ET-1: endothelin-1; CGRP: calcitonin gene-related peptide. ^*∗*^*P* < 0.05 versus WKY. ^#^*P* < 0.05 versus SHR-C.

In SHR-X group, the mRNA expression level of ET-1 was decreased and CGRP was increased significantly compared with the SHR-C (*P* < 0.05, Figure 3), which were consistent with protein expression results. The aorta immunohistochemistry shows the protein expression of ET-1 was decreased and CGRP was increased in SHR-X (*P* < 0.05, Figure 4).

## 4. Discussion

The major findings of the present study are the following. 1. Xinmaitong treatment markedly reduces the arterial blood pressure in SHRs. 2. Meanwhile, the increase in plasma CGRP levels together with upregulation of CGRP mRNA and protein are associated with the decline in ET-1 levels and ET-1 mRNA and protein expression. The present study demonstrates for the first time that Xinmaitong leads to the fall in blood pressure of SHRs and that this antihypertensive effect is, at least in part, due to improvement of arterial tone.

Xinmaitong is a traditional Chinese herbal medicine, which is extracted, concentrated, freeze-dried, and standardized from a mixture of 10 medicinal constituents and has been widely used in the treatment of ASCVD over the recent years [23]. Here, we found that Xinmaitong clearly results in the fall in arterial blood pressure in SHRs, suggesting that compared with western medication therapy traditional Chinese herbal intervention might also probably have a salutary effect on the blood pressure reduction in patient with hypertension.

Accumulating evidence indicates that patients with hypertension are characterized by endothelial dysfunction [24]. ET-1and CGRP are vascular endothelium-derived vasoactive factors. ET-1 has a strong endogenous biological vasoconstrictive effects. Endothelial cell damage is an important mechanism to increase the release of ET-1 [25]. CGRP is a strong endogenous vasodilatory neuropeptides, which has a strong dilation effect on blood vessels. In this study, we investigated the effects of Xinmaitong on ET-1 and CGRP. We found that Xinmaitong can not only improve the blood pressure but also reduce the secretion of ET-1 and promoting release of CGRP. Furthermore, mRNA and protein expression of CGRP and ET-1 were modulated after Xinmaitong treatment in SHRs. We supposed that these alterations are responsible for the vasoactive factors regulation of plasma CGRP and ET-1. The data reported here provide the preliminary evidence to show that Xinmaitong may protect endothelial function by maintaining the balance of vasoconstrictor ET-1and vasodilator CGRP thus helping blood pressure control.

There are some limitations in the present study. Firstly, the exact mechanism underlying Xinmaitong-mediated reduction in blood pressure of SHRs is not clear and beyond the present investigation, which remains to be further elucidated. Second, although our current data suggest that Xinmaitong treatment contributes to the improvement of vasoactive factors, the effect of Xinmaitong on endothelial function also needs to be investigated. Finally, in clinical practice, it is necessary to confirm whether patients with hypertension displayed the fall in blood pressure with Xinmaitong intervention alone.

In summary, the present study for the first time provide data to confirm the beneficial impact of traditional Chinese herbal medicine Xinmaitong treatment where it reduces the blood pressure in SHRs, and this antihypertensive effect might be partly related to the improvement of arterial tone. Further investigation is under way in our laboratory in order to unravel the potential mechanism and clinical application of Xinmaitong in hypertension.

## Figures and Tables

**Table 1:** The hepatic and renal functions in experimental groups.

	Experimental groups		
	WKY	SHR-C	SHR-X
TP (mg/ml)	59.2 ± 11.6	51.0 ± 15.4	46.5 ± 20.3
CREA (*μ*mol/l)	1.6 ± 0.3	1.5 ± 0.4	1.7 ± 0.2
BUN (mmol/L)	7.7 ± 1.1	9.9 ± 3.0	9.0 ± 2.3
TBA (*μ*mol/L)	37.6 ± 5.5	43.6 ± 14.0	35.5 ± 18.6
ALT/GPT (IU/L)	106.9 ± 20.2	103.1 ± 8.9	92.7 ± 7.8
AST/GOT (U/L)	7.7 ± 2.7	7.4 ± 3.1	6.4 ± 6.1

Values are means ± SD. TP: total protein; CREA: creatinine; BUN: blood urea nitrogen; TBA: total bile acid; ALT: alanine transaminase; GPT: glutamate pyruvate transaminase; AST: aspartate aminotransferase; GOT: glutamic oxalo acetic transaminase. WKY: Wistar-Kyoto rats; SHR: spontaneously hypertensive rats; SHR-C: SHR control; SHR-X: SHR Xinmaitong treatment.

## Data Availability

The data used to support the findings of this study are available from the corresponding author upon request.
